# Alginate in Wound Dressings

**DOI:** 10.3390/pharmaceutics10020042

**Published:** 2018-04-02

**Authors:** Blessing Atim Aderibigbe, Buhle Buyana

**Affiliations:** Department of Chemistry, University of Fort Hare, Alice Campus, Eastern Cape 5700, South Africa; 201301441@ufh.ac.za

**Keywords:** alginate, wound dressing, biopolymers, hydrogels, foams, films, nanofibers

## Abstract

Alginate is a biopolymer used in a variety of biomedical applications due to its favourable properties, such as biocompatibility and non-toxicity. It has been particularly attractive in wound healing applications to date. It can be tailored to materials with properties suitable for wound healing. Alginate has been used to prepare different forms of materials for wound dressings, such as hydrogels, films, wafers, foams, nanofibres, and in topical formulations. The wound dressings prepared from alginate are able to absorb excess wound fluid, maintain a physiologically moist environment, and minimize bacterial infections at the wound site. The therapeutic efficacy of these wound dressings is influenced by the ratio of other polymers used in combination with alginate, the nature of cross linkers used, the time of crosslinking, nature of excipients used, the incorporation of nanoparticles, and antibacterial agents. This review provides a comprehensive overview of the different forms of wound dressings containing alginate, in vitro, and in vivo results.

## 1. Introduction

The management of wound remains a challenge despite the progress made so far in the development of wound dressing materials and the level of professional discipline or experience in the field of wound management. Wounds can be classified based on their location, aetiology, nature of the injury, depth, and their appearance [1]. Generally, wounds can be classified as full thickness, partial thickness, and superficial [1,2]. The cost of management of wounds is high and the increasing population size and urban lifestyle is a pointer to the pressing need to develop wound dressings that are effective, appropriate, and affordable. In England, a cost of 184 million pounds was spent on dressing products in 2012 [3]. In the United States of America (USA), an annual cost of 20 billion dollars is spent on the management of chronic wounds [4,5]. The barriers to effective treatment of wounds can be classified as educational, organizational, clinical, and psychosocial [6] (Table 1). Wound dressings are prepared from biopolymers, synthetic polymers and biomaterials. Biopolymers such as chitosan, alginate, fucoidan, hyaluronic acid, etc. are non-toxic, readily available, biodegradable, biocompatible, and non-immunogenic [7,8]. Alginate application in wound dressings is due to its unique properties, such as non-toxicity, biocompatibility, non-immunogenicity, affordability, and high absorption capacity [9]. However, biopolymers are generally limited by their poor mechanical properties. They are combined with synthetic polymers in order to enhance their mechanical properties and tailored to modify their degradation pattern [7,8].

Wound dressings come in different forms and the common problems associated with some of the currently used wound dressings include their inability to maintain a moist environment; poor absorption of wound exudates; delayed wound healing process (i.e., connective tissue synthesis, epidermal migration, and angiogenesis); poor gas exchange between wound and the environment; lack of protection against bacterial infection; difficulty in removal of the wound dressing after healing; and, non-sterile and allergic reactions [10]. The unique properties of alginate make it a potential biopolymer that can overcome the problems in currently used wound dressing by enhancing absorption of wound exudates and minimizing bacterial infections, reducing adverse allergic effects, and improving wound healing because of its biocompatibility, etc. It also exhibits hemostatic properties, which are useful for bleeding wounds [11].

In the design of wound dressings, the important factors that must be taken into consideration are their ability to reduce infections, stop bleeding, absorb exudates, enhance healing and wound debridement, easy to use, biodegradability, easily sterilised, non-toxicity, good water vapour, and gas permeability [12]. Wound dressings can be classified as artificial, traditional, and biomaterial-based dressings [12]. This manuscript will provide a comprehensive overview of the different forms of wound dressings containing alginate, in vitro and in vivo results.

## 2. Wound Healing Process

Wounds can be classified as chronic and acute wounds based on the wound healing process [13]. Chronic wounds are caused by age, obesity, injuries, and chronic conditions, such as diabetes, cancer, etc. Healing of these wounds could take more than 12 weeks [14]. Acute wounds heal between 8–12 weeks and are caused by trauma, such as stabbing, burns, etc. [13,15]. An acute wound can become chronic when there is a failure of the wound to progress via sequential phases of healing, which can be attributed to biofilm bacteria in the wound, resulting in the wound remaining in the inflammatory phase over a prolonged period. The bacteria stimulate the production of proinflammatory cytokines that recruits mast cells neutrophils and macrophages in the wound. The inflammatory cells try to kill the bacteria by secreting proteases and reactive oxygen species (ROS). However, biofilm bacteria are tolerant to antibiotics, ROS, etc., resulting in an elevated level of ROS and proteases that destroy proteins useful for wound healing [16]. Other factors that make acute wound chronic are repeated episodes of tissue injury, resulting in ischemia, such as in pressure ulcer, venous leg ulcers, poor nutrition, underlying disease, excessive friction, immune status, use of inappropriate wound dressing, and bacterial infections [17].

**Figure 1:**
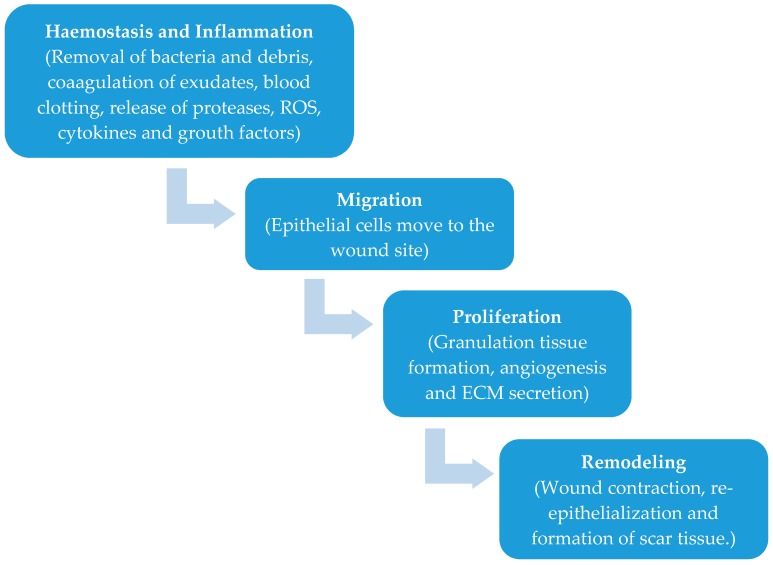
Phases of wound healing.

The process of wound healing is in five phases namely haemostasis, inflammation, migration, proliferation, and remodeling (Figure 1) [4,18,19,20,21,22]. When there is an injury to the skin, haemostasis, and inflammation take place. A major component of the connective tissue of the skin known as fibrinogen helps in the coagulation of the exudates and blood clotting in the wound to stop bleeding [4,13,18,19,20,21,22]. The inflammatory phase occurs simultaneously with the haemostasis phase in which the wound is cleansed of debris and protected from bacterial infection as a result of the release of proteases and reactive oxygen species by the phagocytic cells [4]. The blood monocytes differentiate at the wound site into tissue macrophages releasing growth factors and cytokines recruiting fibroblasts, endothelial cells, and keratinocytes to repair damaged blood vessels [4]. In the migration phase, the epithelial cells move towards the wound site to replace dead cells. In the proliferation stage, the wound is covered completely by epithelium with the formation of granulating tissues. The final phase is the tissue remodeling in which the fibroblasts cover the surface of the wound completely as a new layer of skin. In this phase, there is no evidence of wound and this phase is also referred to as the maturation phase [4,22].

### 2.1. Wound Dressing Classification

**Figure 2:**
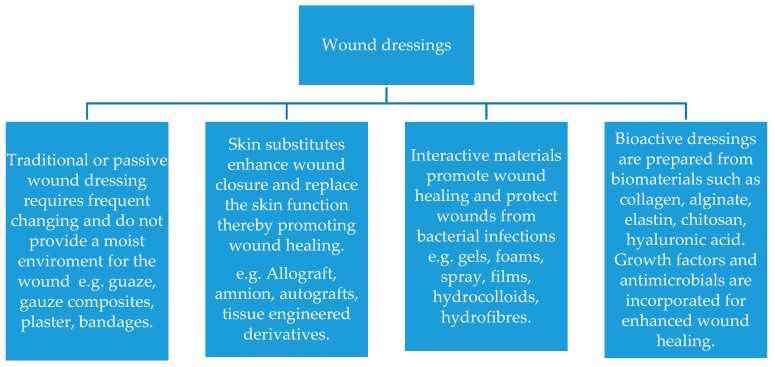
Classification of wound dressings.

Wound dressing can be classified as traditional, biomaterial-based, interactive, and bioactive dressings (Figure 2) [12]. Traditional dressings are also known as passive wound dressings and they are used to protect wounds from contact with the environment and to stop bleeding [12,13]. Examples of the traditional dressings are gauze and gauze-cotton composites that are characterized by high absorption capacity. However, they can cause bleeding, exhibit poor vapor permeation and damage the newly formed epithelium on removal. The leakage of exudates from these dressings can also result in bacterial infections [12]. Biomaterial-based wound dressings can be further classified as allografts, tissue derivatives, and xenografts [12]. Allografts are skin fragments obtained from donors that are either fresh or freeze-dried and their use is limited by immune reaction resulting in rejection by the body. There is also the risk of infection and transmission of diseases [23,24]. They are expensive with limited shelf life [12,23,24]. Tissue derivatives are obtained from collagen but their use is limited by the risk of infections over a long period of usage [12]. Artificial dressings, also known as interactive wound dressings, can be classified as gels, foams, films, spray, composites, etc. [12,13]. They are prepared from biopolymers and synthetic polymers. The most common biopolymers used are alginate, chitosan, gelatin, etc. Wound dressings can also be classified as bioactive wound dressing and examples are alginates, collagens, hydrofibres, and hydrocolloids. Growth factors and antimicrobials are added for improved wound healing process [13]. They are also prepared from biopolymers.

### 2.2. Alginate Properties and Structure

**Figure 3:**
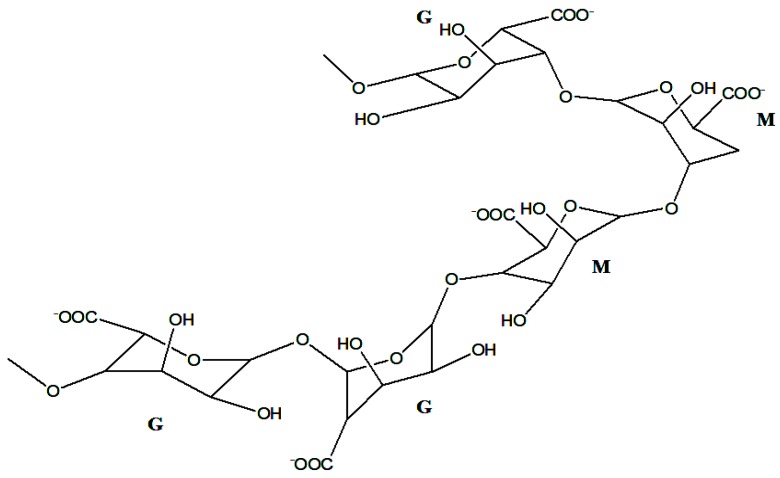
Structure of α-l-guluronic and β-d-mannuronic alginate residues.

Alginate is a biopolymer that is naturally occurring, anionic, and it is obtained from brown seaweed [25]. It readily available, biocompatible, and non-toxic [25]. Wound dressings prepared from alginate are characterized by a moist environment and reduced bacterial infections, which are important factors for wound healing [25]. Alginate is extracted from brown algae (*Phaeophyceae*), *Ascophyllum nodosum*, *Laminaria hyperborea*, *Laminaria japonica*, *Macrocystis pyrifera,* and *Laminaria digitate* [25,26]. It is composed of a varied ratio of guluronate and mannuronate, which is dependent on the source (Figure 3). It is composed of a block of (1,4)-linked β-d-mannuronate (M) and α-l-guluronate (G) residues. The blocks are composed of consecutive G residues (GGGGGG) and M residues (MMMMMM), and alternating M and G residues (GMGMGM). Alginates contain different proportions and the sequence of M and G residues that determine their physical properties and molecular weight [27]. The formation of an alginate-based gel can occur in the absence of temperature. The formation of alginate gels can be achieved by ionic crosslinking with cations or by acid precipitation. The gelation of alginate occurs by the co-operative binding of divalent cations and the G-block regions of the polymer by dimerization of G residues [27]. In the addition of divalent cations such as Ca ions etc. to alginate, the binding of G chains on opposite sides form a diamond-shaped hole, containing a hydrophilic cavity that binds the Ca ions using the oxygen atoms from the carboxyl groups by multi-coordination. This configuration results in egg-box junction zone shape, and this configuration can be achieved using other types of divalent cations. The cations can bind to different block sequence other than G-blocks in alginate [27,28]. The formation of alginate gels can also occur in the presence of trivalent ions such as Al and Fe and the binding of trivalent cations with alginate is more enhanced, resulting in a compact gel network when compared to divalent cations [27,29,30]. The formation of alginate gel can occur by external or internal gelation. These two aforementioned methods differ in how the crosslinking ions are introduced to the alginate polymer. In internal gelation, the exposure of alginate to cations is controlled, resulting in a homogeneous distribution of alginate in the hydrogel. It occurs simultaneously at a number of sites in the polymer. In the external gelation method, there is a diffusion of cations from a region of higher concentration into the interior region of alginate particles, resulting in an inhomogeneous distribution of alginate in the gel matrix network [27,30].

Alginate dressings are prepared by either ionic cross-linking of its solution with calcium, magnesium, barium, lead, cadmium, cobalt, zinc, nickel, manganese, strontium ions, etc., to form a gel, or it can be further processed to form freeze-dried porous sheets in form of foams or fibrous dressings [25,31,32,33]. Alginate dressings can absorb wound fluid in the dry form and form gels that can provide a dry wound with a physiologically moist environment and minimize bacterial infections, thereby promoting rapid re-epithelialization and granulation tissue formation. The immunogenic effect of alginate is influenced by the amount of M-block. Alginate containing a high M-block induces cytokine production when compared to alginate with a high G-block [34]. Proper purification is also essential because the presence of impurities can also induce immunogenic response [34]. Some of the commercially available alginate dressings and their compositions are shown in Table 2 [25,34,35,36,37,38,39,40,41,42,43,44].

## 3. Alginate-Based Wound Dressing

The treatment of acute and chronic wounds is a pressing need and alginate-based wound dressings offer several advantages when compared to the traditional wound dressings. Alginate-based wound dressings exist in the form of hydrogels, films, foams, nanofibers, membrane, and sponges, etc. Alginate dressings absorb wound fluid resulting in gels that maintain a physiologically moist environment and minimize bacterial infections at the wound site. Several researchers have reported the efficacy of these wound dressings.

### 3.1. Hydrogel

Hydrogels are hydrophilic and they have the capacity to absorb a large amount of water [45]. They can be tailored to be chemically stable or degrade when in contact with the biological fluids over a period of time. Hydrogels have been employed for wound healing applications because of their biocompatibility, ability to load and release bioactive agents, porosity, high water content, and flexibility [46,47,48]. However, despite the unique properties of hydrogels that make them useful in wound dressing, they also suffer from some shortcomings, such as poor mechanical stability at swollen state, ability to dehydrate if not covered, suggesting the need for a secondary dressing, and their ability to cause skin maceration, making them difficult to secure [49,50]. In order to overcome the poor mechanical stability of hydrogels, they are prepared by the combination of synthetic and natural polymers, resulting in hydrogels with good mechanical properties [49]. Sodium alginate-based hydrogels have been reported by several researchers.

Saarai et al. prepared hydrogels by combining sodium alginate (SA) and gelatin (G) (Type B, 250 Bloom) in a ratio of SA/G 70/30, 60/40, 50/50, 40/60, and 30/70 as potential wound dressings [51]. The ratio of sodium alginate and gelatin influenced the morphology of the hydrogels. Hydrogels with the composition of SA/G 70/30, 60/40, and 50/50 exhibited droplet morphologies, while hydrogels 40/60, 30/70, and 20/80 exhibited fibrous morphology. Their high water content and swelling behavior was influenced by their composition and revealed their potential application as exudative wound dressings. The hydrogels water uptake was 72.52% for SA/G 70/30, 81.86 for 60/40 SA/G, 84.03% for SA/G 50/50, 87.97% for SA/G 40/60, and 90.1% for SA/G 30/70, respectively. The high swelling ability of the hydrogels was attributed to the hydrophilic functional groups such as NH_2_– and COO– from the biopolymers [51]. In another research report by Saarai et al. alginate-gelatin hydrogels with different ratios of sodium alginate and gelatin SA/G 20/80, 30/70, 60/40, 50/50, 40/60, 30/70, 80/20 were prepared [52]. The hydrogels were crosslinked by using either calcium chloride or glutaraldehyde. The water uptake of the hydrogels decreased with increase in the concentration of the crosslinker used, suggesting high mechanical strength. The equilibrium swelling of the hydrogels also decreased with increase in the content of gelatin in the hydrogels. The crosslinking time of the hydrogels influenced their mechanical properties. Short crosslinking time resulted in hydrogels with poor mechanical strength, which was characterized by a rapid increase in the swelling of the hydrogels in the first 4 h, followed by a collapse of hydrogel network structure. However, increasing the crosslinking time resulted in a decrease in the swelling capacity of the hydrogels because of the formation of a higher density of crosslinking points in the network structure. The hydrogel with a ratio of 50:50 sodium alginate:gelatine was very appropriate for wound dressing and it was characterized by good swelling ability at selected pH values [52]. Straccia et al. coated alginate hydrogels with chitosan [53]. The gels were prepared by internal gelation method. The hydrogel exhibited good antibacterial activity against *Escherichia coli.* The hydrogels were characterized by high water uptake of 200–450% and good homogeneity. These findings suggested that the hydrogels would prevent the wound bed from accumulating exudates and protect the wound from excessive dehydration. Stability in normal saline solution increased upon coating the hydrogels with chitosan. Cryo-SEM (Cryogenic scanning electron microscopy) analysis of the coated hydrogels indicated a regular and compact surface with altered internal morphology. Coated hydrogels exhibited antibacterial activity against *Escherichia Coli.*, and cytotoxicity tests demonstrated that chitosan hydrochloride did not elicit any acute toxic effects on the mesenchymal stromal cells. Release studies using rhodamine B as a low molecular weight hydrophilic drug showed that coating the hydrogels reduced the drug release kinetics, resulting in a sustained drug release [53]. Incorporating bioactive agents and nanoparticles onto alginate-based hydrogels have also been reported to enhance wound healing process.

Yu et al. developed wound dressings composed of alginate hydrogel and simvastatin-incorporated in mesoporous hydroxyapatite microspheres. The wound dressings enhanced the formation of new blood vessel and enhanced re-epithelialization of the cutaneous wounds in vivo [54]. Incorporated simvastatin enhanced angiogenic differentiation of human umbilical vein endothelial cells in vitro. It also enhanced the expression of hypoxia-inducible factor-1α and vascular endothelial growth factor, which are important in angiogenesis [54]. Mohandas et al. prepared alginate hydrogel/zinc oxide nanoparticles composite bandage wound dressing with interconnected pores in the size range of 200–400 μm with a porosity of 60–70% [55]. The presence of zinc oxide reduced the swelling ratio of the wound dressing to 16–20. After two weeks, no significant change in the swelling ratio of the wound dressing was observed. The presence of zinc oxide nanoparticles also changed the degradation nature of the composite bandages by 10–15% after one week and by 30–40% after three weeks of immersion in PBS containing lysozyme. The antibacterial activity of the wound dressing against *S. aureus* and *E. coli.* reduced with a decrease in the amount of zinc oxide nanoparticle in the wound dressing. The wound dressing was also effective against *C. albicans.* In vivo evaluation revealed re-epithelialization after 48 h revealing that the zinc oxide nanoparticles enhanced the proliferation and migration of keratinocyte cells to the wound site [55]. Singh et al. prepared hydrogels from a combination of polyvinyl pyrrolidone and alginate, followed by incorporation of silver nanoparticles using gamma radiation [56]. The percentage composition of polyvinyl pyrrolidone was 10% and 15%, while the percentage of alginate was 0.5% and 1%. Different doses of gamma irradiation of 25 and 40 kGy were employed. The fluid uptake of the hydrogels was in a range of 1881–2361% over a period of 24 h. The hydrogels containing 70 ppm nanosilver exhibited good antimicrobial activity [56]. The fluid absorption capacity of the hydrogels revealed their potential application for exuding wounds. Nazeri et al. prepared honey-based alginate hydrogel with a thickness of 3–4 mm and good transparency [57]. In vivo evaluation of the hydrogel on Wistar rat revealed that the time taken for wound healing was seven days when compared to alginate hydrogels without honey that required eight days for wound healing. The finding revealed that the presence of honey in the hydrogel stimulated angiogenesis and the growth of fibroblasts as a result of the delivery of hydrogen peroxide. The hydrogel enhanced re-epithelialization in the wounds which was evident by the thickness of the epiderm. The wound healing of the hydrogels revealed the synergistic effect of the alginate hydrogel and honey [57]. Combining anticancer drug with alginate-based hydrogels and incorporation of antimicrobial agents onto alginate-based hydrogels resulted in enhanced re-epithelization in vivo.

Murakami et al. prepared hydrogel sheet containing blended powder of alginate, chitin/chitosan, and fucoidan for wound dressing [58]. The hydrogel protected the wound by providing a moist environment for an enhanced healing process in full-thickness skin defects on rats. Granulation tissue and capillary formation in the healing-impaired wounds treated with the hydrogel and mitomycin C was significant on day seven. However, the degree of re-epithelialization was reduced, suggesting that the hydrogel hindered the migration of keratinocytes [58]. The hydrogels exhibited a minor healing effect on wounds that were not treated with mitomycin C. The wound dressing with the diameter of 10 mm absorbed 0.3 g of exudate from mitomycin C-treated wounds over a period of two days. The wound dressing was easily applied and removed from the wound. The hydrogel absorbed substances involved in the wound healing process such as the growth factors and cytokines from the blood plasma or exudate in the wound, thereby stimulating wound healing process [58]. Kamoun et al. prepared poly(vinyl alcohol)-sodium alginate hydrogel membranes loaded with sodium ampicillin for wound dressing application by freeze-thawing cycle method [59]. The percentage weight content of sodium alginate was in the range of 0–75% *w*/*w* and poly(vinyl alcohol) was 10% *w*/*v*. Increasing sodium alginate content to 75% resulted in an increase in the water uptake by 4200%. The hydrogel with 75% content of sodium alginate exhibited a weight loss of 60%, while hydrogels membranes without sodium alginate exhibited 18% weight loss indicating that an increase in the content of sodium alginate in the hydrogel membranes enhanced the hydrolytic degradation of the membranes, significantly. The maximum tensile strength and elongation of the hydrogel membranes decreased with an increase in the content of the sodium alginate. The pore size and distribution also increased with increase in the content of the sodium alginate. The hydrogel membrane wound dressing system containing ampicillin exhibited sufficient antibacterial activity and were found to be suitable for wound dressing [59]. Preparation of hydrogels with good swelling capacity from the combination of alginate with other polysaccharides has been reported by some researchers.

Xing et al. prepared alginate-chitosan hydrogels for wound dressing [60]. An increase in the concentration of alginate decreased the swelling capacity, but increased the water holding capacity by 80%. The hydrogel was non-toxic to the cells [60]. Rudyardjo and Wijayanto prepared hydrogel chitosan-alginate using a plasticizer, lauric acid [61]. The addition of lauric acid enhanced the mechanical properties of the hydrogel. The concentration of lauric acid used was 0%, 1%, 2%, 3%, 4%, and 5% *w*/*v* and the composition of chitosan-alginate was 4:1 (*v*/*v*). Hydrogels prepared using 4% lauric acid exhibited a tensile strength of 9.01 ± 0.65 MPa, a thickness of 125.46 ± 0.63 µm, absorbed liquids 601.45 ± 1.24%, and elongation of 28.89 ± 1.01%. Increasing the lauric acid by more than 4% resulted in a hydrogel with a decreased capacity to absorb water. The mechanical properties revealed the suitability of the hydrogels for wound dressing. Increasing the lauric acid plasticizer used in preparing the hydrogels by more than 1% resulted in an increase in the thickness, elongation, and water absorption capacity of the hydrogels, with a decrease in the tensile strength [61]. Devi et al. prepared hydrogels from a combination fibrin, chitosan, and sodium alginate in varied ratio [62]. Hydrogel containing 4% *w*/*v* fibrin, 0.1% *w*/*v* chitosan, and 0.2% *w*/*v* sodium alginate exhibited good mechanical properties of 0.35 ± 0.13 thickness, 4.44 ± 0.34% elongation and tensile strength of 23.34 ± 1.04 MPa. The hydrogel exhibited fibrous morphology with pore sizes of 100–400 mm suggesting that the hydrogel has a potential to prevent bacterial infection, enhance wound healing, and favourable for cell attachment and skin formation [62]. Zhou et al. prepared hydrogel composed of gelatin, sodium alginate, and hyaluronic acid by a freezing-drying method using 1-ethyl-3-(3-dimethyl aminopropyl) carbodiimide as a crosslinker [63]. The ratio of the gelatin, sodium alginate, and hyaluronic acid in the hydrogels were 1:8:1, 3:6:1, 4.5:4.5:1, 6:3:1, and 8:1:1. The ratio of the polymers had no effect on the surface and cross-section morphologies of the hydrogels. Sodium alginate in the hydrogel matrix enhanced the water vapor transmission capacity of the hydrogel suggesting their potential application as wound dressing materials due to their ability to provide a moist environment for comfortable wound healing. However, the difference in the water vapor transmission rate of the hydrogels was not significant [63].

Factors that influenced the swelling capacity of alginate-based hydrogels are the composition of polymers used [52,56,57,58,59,60,61,62,63], the plasticizer [61], and the presence of nanoparticles [55]. Their good swelling capacity is a good feature for wound dressings because it reveals their potential to keep the wound environment moist, thereby reducing the risk of bacterial infection, thereby enhancing the wound healing process [25]. The antibacterial activity of the hydrogels was enhanced by the incorporation of antibacterial agents [54,59], combining the hydrogel with an anticancer agent [58], incorporation of nanoparticles [55,56], and the coating of the hydrogels with honey [57] and chitosan [53].

### 3.2. Films/Membranes

**Figure 4:**
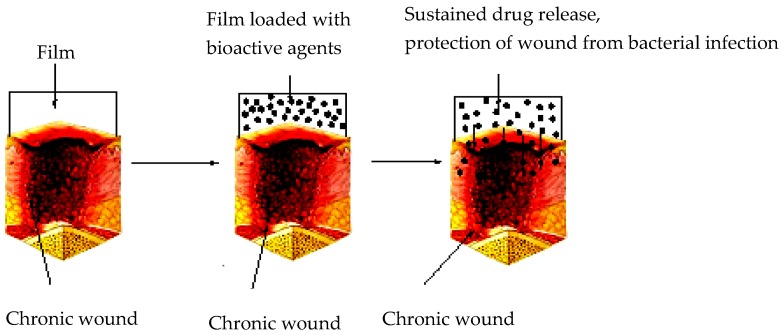
Films for wound dressing.

Sodium alginate-based films have been designed and reported by some researchers as potential wound dressing materials (Figure 4). Films are useful for wound dressing. They enhance wound healing process, permeability to water vapor, carbon dioxide and oxygen, protect the wound from bacterial infections, and their mechanical properties can be improved by combining alginate with other polymers. However, they are not effective wound dressing for wounds with excessive exudates [10,11]. Sodium alginate films incorporated with essential oils were developed by Liakos et al. [64]. Essential oils, such as chamomile blue, cinnamon, lavender, tea tree, peppermint, eucalyptus, lemon grass, and lemon oils were incorporated onto the films and glycerol was used as a plasticizer [64]. Igepal surfactant was used in order to enhance the dispersion of the oil in the film matrix. The addition of the surfactant enhanced the miscibility between the essential oil and the matrix. The films exhibited good antimicrobial activity. The films containing tea tree, cinnamon, lemon grass, and peppermint were able to inhibit *C. albicans* at all of the concentrations selected and inhibited *E. coli*. The film containing elicriso oil was effective against *C. albicans* only [64]. The antibacterial activity of the films was selective and influenced by the nature of essential oil loaded onto the films. Pereira et al. prepared hydrogel films composed of alginate and *Aloe vera* gel in varied proportions of 75:25, 85:15, and 95:5 *v*/*v*. Their mechanical properties were suitable for skin applications and their swelling increased with increase in the amount of *Aloe vera* in the films [65]. The films were characterized by a smooth surface, high malleability, and thicknesses in a range 66.14–69.00 μm [65]. The non-crosslinked films exhibited tensile strength and elongation values at break point, in a range of 21.44–40.44 MPa and 5.94–13.27%, respectively. Addition of glycerol resulted in a significant decrease in the rigidity, which was evident by a decrease of the tensile strength in the range of (40.44–28.66 MPa), and an increase in the elongation at break in the range of (5.94–13.27%). The aforementioned behavior was attributed to the plasticizing effect of glycerol that decreased the polymer-polymer interactions and increased the mobility of the polymeric chains. The addition of *Aloe vera* decreased the tensile strength and elongation at break of the films. Incorporating 5% of *Aloe vera* did not have a significant influence on the water uptake of the films. Incorporating 15% and 25% of *Aloe vera* onto the films increased the water uptake, significantly due to the hydrophilic properties of *Aloe vera* [65]. In another research report, Pereira et al. sodium alginate films containing *Aloe vera* extract were prepared by casting/solvent evaporation technique [66]. The thermal and mechanical properties of the films were influenced by the loaded *Aloe vera* extract which revealed their potentials as wound dressing materials. The film thickness was in the range of 29.00 ± 2.80 µm and 39.33 ± 4.58 µm. The incorporation of *Aloe vera* at a concentration of 12% resulted in an increase in the film thickness. Blending alginate with *Aloe vera* caused a slight increase in the degradation temperature of the films, suggesting that a strong polymer-polymer interaction was visible from incorporating *Aloe vera* [66]. Alginate-based films incorporated with antibacterial and selected bioactive agents have also been reported by some researchers.

George et al. prepared films containing gelatin and sodium alginate by a solvent casting method, followed by the loading of silver sulphadiazine [67]. The films were biodegradable and were able to entrap 93–97% of silver sulphadiazine. The release profile of silver from the film was sustained over a period of seven days. The folding capability of the films was in the range of 337 ± 0.96–459 ± 0.69, indicating their ability to withstand the mechanical pressure and good flexibility. The percentage drug encapsulation in the films was in a range of 90.23–97.75%, which are useful for wound dressing [67]. Li et al. developed strontium loaded silk fibroin-sodium alginate films for wound dressing [68]. The quantity of strontium loaded onto the films was in the range of 1–30 mg/mL. The water absorption rate of the strontium-loaded films decreased with an increase in the content of strontium. Films that do not contain strontium exhibited water absorption rate of 900%, while the absorption rates of the films with 1 mg/mL and 30 mg/mL of strontium were 800% and 100%, respectively. These findings revealed that the films become more hydrophobic after incorporating strontium. The water vapor transmission rate for the films was in the range of 2000–2700 g·m^−2^·day^−1^, which is sufficient to avoid excess exudates around the wound site that can cause bacterial infections and retards wound healing. 

The ultimate tensile stress, tensile strain and E-modulus of the films were in the range of 1.38 ± 0.01–35.65 ± 4.92, 108.83 ± 10.87–47.10 ± 8.26, and 6.45 ± 1.23–99.97 ± 13.95, respectively. The cumulative release of strontium from the films containing 1 mg/mL, 5 mg/mL, 10 mg/mL, and 30 mg/mL strontium over a period of 72 h were 0.659 mg/mL, 0.989 mg/mL, 1.208 mg/mL, and 1.372 mg/mL, respectively. Films incorporated with 5 mg/mL strontium induced high amounts of bFGF (basic fibroblast growth factor) and VEGF (Vascular endothelial growth factor) over a period of four days in vitro, indicating it can induce vasculogenesis benefits in wound dressings [68]. Rezvanian et al. reported composite film of sodium alginate blended with pectin or gelatin [69]. From the pre-formulation studies, sodium alginate blended with pectin exhibited superior mechanical properties. The films were non-toxic and alginate/pectin composite film loaded with simvastatin was found to be a suitable and potential wound dressing [69]. The water vapor transmission rates for sodium alginate film loaded with simvastatin, sodium alginate-pectin film loaded with simvastatin, and sodium alginate-gelatin film loaded with simvastatin were 1455, 1385, and 1394 g/m^2^/day, respectively, indicating the permeability of the films to water vapor and their ability to maintain moist environment on the wound site in exudative wounds without excessive dehydration. The apparent viscosity of sodium alginate-gelatin film loaded with simvastatin was 250 s^−1^, which was significantly reduced when compared to other gels. The rate of drug release from the film was influenced by the ratio of the polymers. The release of the drug from sodium alginate-pectin film loaded with simvastatin, sodium alginate loaded with simvastatin, sodium alginate-gelatin loaded with simvastatin was 0.826 ± 0.069 mg/cm^2^, 0.929 ± 0.101 mg/cm^2^, and 1.736 ± 0.134 mg/cm^2^, respectively. In vitro cytotoxicity evaluation on normal human dermal fibroblast cells revealed cell viability of 83.20% with respect to untreated cells [69]. Thu et al. developed a bilayer sodium alginate-based hydrocolloid film as a slow-release wound healing device [70]. The bilayer was encapsulated with ibuprofen in the upper layer with a drug-free lower layer. The bilayer films exhibited superior rheological and mechanical properties of 27.22 ± 0.95 MPa tensile strength and 59.02 ± 2.54% elongation when compared to the single layer films with 20.82 ± 2.29 MPa tensile strength and 23.74 ± 3.30% elongation. The time required for diffusion of the water molecules across the bilayer films with the thickness of 3.14 ± 0.04 mm was longer when compared to single layer films with lower thickness (0.69 ± 0.02 mm). The release of ibuprofen liberated from bilayer film was 0.021 ± 0.05 mg/cm^2^/h and slower when compared to the single layer films 0.036 ± 0.005 mg/cm^2^/h, indicating that the presence of a bilayer film act as a controlling membrane. The lower layer of bilayer film retained moist in the wound thereby accelerate epithelialization. In vivo studies of wounds treated with bilayer films were covered with epidermis and newly formed dermis. Wounds treated with single layer films showed re-epithelialized epidermis composed of fewer keratinocytes on the surface [70]. Alginate-based films have also been used in combination with laser therapy and metal-based nanoparticles, resulting in enhanced wound healing in vivo.

Dantas et al. reported the application of sodium alginate-chitosan based films for burns in combination with laser therapy [71]. In vivo studies on male rats using a combination of laser therapy with the films enhanced rapid replacement of type III for type I collagen, blood vessels formation, epithelization, collagenization, and promoted good arrangement of the newly formed collagen fibres. The combination improved the burn healing significantly resulting from the anti-inflammatory activities of chitosan and alginate that potentiate low-level laser therapy biostimulatory activity [71]. Sharma et al. prepared silver nanoparticle-sodium alginate-chitosan films with varied ratio [72]. The film with a 1:1 sodium alginate-chitosan films ratio exhibited a higher % water uptake, superior mechanical properties, easy to handle, and smoother surface when compared to other films. The thickness of the 1:1 ratio film was 5.562 ± 0.43, the elongation value at break was 23.80 ± 2.53%, the tensile strength was 16.55 ± 3.62 MPa with Young Modulus of 520 ± 88.82 MPa, which was high when compared to other ratios. The films were also effective at inhibiting Gram-positive bacteria which further revealed their potential in wound dressing [72].

### 3.3. Nanofibres

Nanofibres prepared from sodium alginate have been reported by few researchers. They are potential materials for wound dressing. Nanofibres mimic the extracellular matrix, thereby enhancing the proliferation of epithelial cells and the formation of new tissue. [73,74] Their nanometer diameter and nanofibrous meshes promote haemostasis of injured tissues, enhance fluid absorption, promotes dermal drug delivery, cell respiration, and high-gas permeation, thereby preventing bacterial infections [73,74]. However, the method of preparation influences the outcome of the nanofibers. Controlling pore structure of the nanofibers is difficult when using electrospinning technique. Using self-assembly method of preparation results in nanofibers with shapes that are not uniform. Drug loading onto the nanofibers is low when fiber mesh or self-assembly is used for drug loading process [75,76].

The addition of nanoparticles onto nanofibres promotes their antibacterial activity. Shalumon et al. prepared sodium alginate-poly (vinyl alcohol)-zinc oxide nanoparticles nanofibers by electrospinning technique [77]. The nanoparticles size was 160 nm and different concentrations, namely: 0.5%, 1%, 2%, and 5% were used in the preparation of the films. Nanofibers containing 0.5% and 1% zinc oxide nanoparticles were less toxic when compared to those with a higher percentage of the nanoparticles. The nanofibers were effective against *S. aureus* and *E. coli.* due to the presence of the nanoparticles [77]. In the preparation of the nanofibers, the ratio of alginate used can result in structural defects.

Hu et al. reported nanofibers electrospun from sodium alginate and polyethylene oxide [78]. The spinnability of the alginate solution into nanofibers was possible by the addition of polyethylene oxide. The ratio of sodium alginate and polyethylene oxide used to prepare the nanofibers were 1:3, 1:1, 3:1, and 0:1. The smoothness and the diameter of the nanofibers were influenced by the ratio of sodium alginate and polyethylene oxide used for the preparation. Increasing the ratio of alginate in the 3:1 nanofibres resulted in spindle-like defects and decreased diameter. Increasing the alginate content up to 50% (i.e., 1:1 nanofibre) resulted in the formation of smooth nanofibers with an average diameter of 105 nm. The tensile strength of the nanocoated hybrid yarn was superior to the uncoated yarn [78]. The antibacterial and wound healing effects of essential have been employed in alginate-based nanofibers.

Hajiali et al. prepared sodium alginate-polyethylene oxide nanofibers loaded with lavender (an essential oil) with an average diameter of 93 ± 22 nm [79]. The electrospinnability of the solution of alginate was made possible by the use of polyethylene oxide and Pluronic F127. The prepared electrospun mats were characterized by bead-free nanofibers, with an average diameter of (91 ± 21) nm and (93 ± 22) nm in the sodium alginate-polyethylene oxide and sodium alginate-polyethylene oxide-lavender oil nanofibers, respectively. The difference in diameter was not statistically significant (*p* > 0.05), revealing that the addition of lavender oil did not affect the morphology of the nanofibres. Contact angle measurements further revealed that both nanofibers prepared were highly hydrophilic, with an apparent water contact angle of 21 ± 2° and 26 ± 2° for sodium alginate-polyethylene oxide and sodium alginate-polyethylene oxide-lavender oil nanofibers, respectively, indicating the ability of the nanofibers to absorb wound exudates. The tensile strength of sodium alginate-polyethylene oxide and sodium alginate-polyethylene oxide-lavender oil nanofibers was 13 ± 2 MPa and 9 ± 1 MPa, respectively. The elongation at break was 2.8 ± 0.4% and 1.6 ± 0.2% for the sodium alginate-polyethylene oxide and the sodium alginate-polyethylene oxide-lavender oil, respectively. Their good mechanical properties indicate that the nanofibers are flexible, can be easily handled without breaking, and can adapt to skin wounds. Sodium alginate-polyethylene oxide-lavender oil was effective against *S. aureus* when compared to sodium alginate-polyethylene oxide nanofibers that did not inhibit bacterial growth. In vivo studies of the nanofibers on burns revealed a complete disappearance of erythema within 48 h, which indicate that the nanofibers ability to prevent inflammation induced by UVB irradiation. The levels of IL-6, IL-1β, and TNF-α were up by 4, 10, and 7 times, respectively, but the levels were lower when compared to the UVB-irradiated group of animals that were not treated with the dressing. After 48 h, the anti-inflammatory effect of the nanofibers was significant in which the levels of IL-6, IL-1β, and TNF-α for the treated animals were up to 7, 24, and 19 times lower than those for the untreated animals, respectively. The nanofibers were important in suppressing the production of cytokines and their anti-inflammatory activity was enhanced by the addition of lavender essential oil in the nanofibres. Furthermore, when comparing the nanofibers with the commercially available alginate-based dressing (Tegaderm™) also revealed that the inhibitory effect of the electrospun nanofibers on cytokine production was higher than Tegaderm™ after the first 6 h from the UVB exposure [79]. Alginate-based nanofibers have been prepared using synthetic polymers, such as poly(vinyl alcohol) and poly(ethylene oxide).

Üstündağ et al. evaluated the potential of electrospun nanofibrous mats as wound dressings [80]. The nanofibers were prepared from sodium alginate and poly(vinyl alcohol). Poly(vinyl alcohol) solution with a concentration of 9% and sodium alginate solution with a concentration of 1% were blended in the volume ratio of 2/1. The nanofibers were characterized by a uniform and continuous fiber formations with fiber diameter of 100.35 ± 12.79 nm. The nanofibers air permeability effect of 0.14 ± 0.02 m^2/^m^3^/min was poor and this was because of the process parameters. In vivo studies on rabbits revealed that the percentage wound contraction ability of the nanofiber on the 15th day was 98%, which was comparable to Suprasorb-A, a commercially available nanofibre with a percentage wound contraction of 99% [80]. Coşkun et al. prepared similar poly(vinyl alcohol)/sodium alginate nanofibrous mats as a wound dressing material. The nanofiber revealed enhanced wound healing characterized by re-epithelization, vascularization and formation of hair follicles. The nanofibrous mat exhibited healing performance when compared to other wound dressings. The nanofiber acted as an artificial skin on the wound region till the formation of a new tissue [81]. Park et al. prepared sodium alginate nanofibers by blending with poly(ethylene oxide) and lecithin. The ratio of sodium alginate and poly(ethylene oxide) was 1/1, 2/1, 3/1, 1/ 2, 2/2, 3/2, *w*/*w* with 0.3 *wt*% lecithin. In the blend, the conductivities were reduced when increased amount of poly(ethylene oxide) was added because of the low viscosity of poly(ethylene oxide) compared to sodium alginate. Reduced content of poly(ethylene oxide) in the blend resulted in beaded fibers as in the case of 2/1 nanofibers. Immersion of the nanofiber in deionized water and phosphate buffer solution indicated the ability of the nanofibers to absorb exudates [82]. Antibacterial agent has been incorporated onto alginate-based nanofibres.

Fu et al. prepared sodium alginate-poly(vinyl alcohol)-moxifloxacin hydrochloride nanofibrous membranes [83]. The nanofibers were prepared by electrospinning by blending 12% poly(vinyl alcohol) and 2% sodium alginate in a ratio of 8:2, 7:3, 6:4, 5:5, and 4:6. The water vapor permeability of the nanofibers was (11.85 ± 3.01) × 106. The diameter of the nanofiber was 175 ± 75 nm. Increasing the amount of moxifloxacin hydrochloride did not increase the diameters of the nanofibres. The % swelling of the nanofibers was 108 ± 6.45% and the percentage weight loss was 4.94 ± 1.98%. The antibacterial activity of the nanofibers increased with increase in the concentration of moxifloxacin hydrochloride in the nanofiber. In vivo studies of the nanofibers in rats with full-thickness round wounds with a surface area of 0.79 cm^2^ revealed that on day 8 and 14, healing was significant. The nanofibers also exhibited good antibacterial activity against *S. aureus* and *P. aeruginosa* [83].

### 3.4. Foams

Foams are solid porous matrices that can be sterilized and administered to wounds without causing discomfort to the patient. However, if parameters such as thickness, density, and mechanical properties, such as tensile strength and elongation of the freeze-dried foam, are not properly tailored, their application in wound dressing can cause discomfort to the patients and maceration of skin around the wound periphery [84]. Foams can absorb exudate, protect the wound from maceration, enhance gaseous exchange, and can provide a moist environment for the wound. Wounds that foam dressings are applicable are traumatic wounds, burns, diabetic ulcers, etc. Foam dressings can also be used as a secondary dressing [85]. Alginate-based foams are characterized by extended hydration time, they can be removed from wound site without severe damage to the tissue. However, their use requires frequent dressing and they are not suitable for wounds with low exudates, and dry wounds [10]. Alginate-based foams that have been reported were prepared by loading antibacterial agent.

Hegge et al. loaded curcumin to alginate foams for the treatment of infected wounds [86]. The foam loaded with curcumin exhibited extended hydration time. The release of curcumin from the foam was sufficient upon hydration, resulting in 100% curcumin-mediated phototoxicity of viable *E. faecalis* cells in vitro. However, *E. coli* was less susceptible to photokilling with curcumin loaded foams and was influenced by the solubilizers of curcumin used in the foams. Only foams containing PEG 400 as the solubilizer of curcumin-induced inactivation of 81% (∼29 J/cm^2^) of the viable *E. coli.* cells. The foams hydrated rapidly when in contact with the physiological solution and remained intact after the release of the loaded curcumin, suggesting that they can be removed from the wound site without tissue damage prior to irradiation, thereby minimizing light attenuation in a photodynamic therapy (PDT) [86]. Valerón et al. reported alginate foams for antibacterial photodynamic therapy of infected wounds. The foams were loaded with 5,10,15,20-tetrakis(4-hydroxyphenyl)porphyrin. The foams were prepared from poloxamer (Pluronic F127) and β-cyclodextrin derivatives. The foams were thin and flexible and easy to handle. The percentage of drug loaded onto the foam was between 0.12–0.13% *w*/*w*. The release of the drug was influenced by the presence of β-cyclodextrin derivatives [87].

### 3.5. Topical Formulation

Topical formulations are semi-solid dosage and occur in the form of gels, ointment and creams [88]. It offers several advantages such as patient compliance, incorporated drugs are delivered more selectively to a target site and it overcomes fluctuation in the levels of drug [88]. Skin is the ideal site for the delivery of drug substances for systemic and local effects. The epidermal and dermal of the skin act as a barrier that inhibits drug transport. Selecting the right excipient can result in reduced surface adsorption, immunogenic effect, and overcome degradation [89]. Preparing the formulation is very important because it affects the drug delivery, integrity of the dosage, and duration of the efficacy of the active agent [89]. Loading the right bioactive agent is very important because its interaction with the excipient influences its permeation effect, its stability, its rate of metabolism, and the rate of drug delivery [89]. There is very little research report on sodium alginate-based topical formulation for wound dressing. However, the application of topical formulation for wounds is limited by reduced penetration in open wounds, risk of hypersensitivity reactions, systematic absorption in large wounds, contamination of the formulation from day-to-day bodily contact because of frequent applications and alteration of normal cutaneous flora [89].

Sodium alginate has been employed in topical formulation for wound healing. Ahmed et al. prepared topical formulation from chitosan and sodium alginate loaded with fucidin or *Aloe vera* with vitamin C using carbopol 934p as a gelling agent [90]. The drug content of the gels was in the range of 94.75–99.83%. The gels exhibited good viscosity in the range of 3246.31–7600.12 and spreadability of the gels were in the range of 12.21 to 28.13 gm·cm/s. The formulation containing 1% *w*/*w* sodium alginate and 0.5% *w*/*w* reduced 90.4% of the wound area and 94.65% of the wound area after the 12th day, respectively in vivo [90].

### 3.6. Wafers

Lyophilized wafers are prepared by freeze-drying polymer solutions resulting in solid porous structures that can be applied to exuding wound surfaces [91]. Their physical structure is similar to foam wound dressings. They also act as drug delivery systems and can be loaded with bioactive agents that can enhance wound healing process. They absorb wound exudates and transform into a gel and provide a moist environment useful for wound healing. Their preparation process is very important because poor preparation process from poor ratios of materials can result in rigid, sticky, and non-porous wafers that are not suitable for wound dressing [92]. Wafers loaded with silver sulfadiazine have been reported.

Boateng et al. prepared lyophilized wafers comprising sodium alginate and gelatin of varied ratios of 0/100, 75/25, 28 50/50, 25/75, and 0/100 for sodium alginate and gelatin, respectively [93]. It contained 0.1% *w*/*w* silver sulfadiazine. Formulation with the absence of sodium alginate was characterized by a non-porous morphology. The addition of sodium alginate in the formulation resulted in uniform pores in the polymeric network. The water uptake of the wafers was also influenced by the presence of sodium alginate. The wafers containing only sodium alginate exhibited a high water uptake of 2299.79 ± 151.29%. Increasing the content of gelatin in the hydrogels decreased the swelling capacity of the hydrogels. The drug entrapment efficacy of the wafers was 80% for 25/75 sodium alginate/gelatin and 93% for 75/25 sodium alginate/gelatin [93]. Formulations containing a higher amount of sodium alginate released the loaded drug faster when compared to other formulations over a period of 7 h. The high swelling ability, mechanical properties, and drug release profiles suggest that sodium alginate-based wafers are potential wound dressings for highly exuding wounds [93]. Wafers have also been prepared from some biopolymers such as carrageenan, xanthan gum, guar gum, etc. 

Matthews et al. prepared a class of wafers from sodium alginate, xanthan gum, and methylcellulose. The wafers absorbed water instantaneously resulting in a transformation from a glassy state to viscous gels. The presence of sodium alginate in the formulations enhanced their swelling ability and revealed the potential of the gels in wound dressing [94]. Pawar et al. developed wafers from polyox, carrageenan and sodium alginate [95]. The wafers were loaded with streptomycin and diclofenac for the treatment of chronic wounds. The drug loaded wafers were flexible, soft, and non-brittle. The porosity of the wafers was enhanced by annealing. Their flexible nature protected the newly formed skin tissue in the wound from damage. The release profile of the drugs from the formulation was controlled revealing their potential to protect the wounds from bacterial infection and reduce pain associated with injury. However, sterilization of the wafers by steam can result in structural collapse due to moisture. Gamma irradiation is the suitable means of sterilization of the wafers at appropriate dose [95]. Gowda et al. prepared wafers containing sodium alginate and guar gum. The excipient used did not alter the efficacy of the loaded drugs. The average diameter of the wafer was found to be 430 µm and the morphology of the wafers was characterized by an interconnecting, porous network and a spongy-like structure. The viscosity of the formulation was in the range of 3.79–14.08 Pa·s. The water uptake capacity of the formulations was in a range of 323.2 ± 59.47–509.7 ± 67.23%, indicating that the lyophilized wafers can absorb and retain a substantial amount of water. In vivo studies performed on Wistar rat over a period of 14 days revealed a decrease in the diameter of the wound when treated with the drug loaded wafers [96].

## 4. Conclusions

Alginate-based dressings exist in different forms, such as hydrogels, films, foams, nanofibers, and in topical formulations. Their use in these wound dressings has been characterized by high water uptake, increased porosity, sustained drug release, and non-immunogenic effects. The aforementioned properties enhance rapid re-epithelialization, granulation tissue formation, and wound healing. In hydrogels containing sodium alginate, their swelling capacity was influenced by the nature of crosslinker, ratio of polymers used affected the pore sizes of the matrix network, and the addition of nanoparticles. Nanoparticles, such as zinc oxide and nanosilver, also altered the degradation pattern of the hydrogels, enhanced the antibacterial activity of the hydrogels, and promoted the proliferation and migration of keratinocyte cells to the wound area. Addition of bioactive agents to the hydrogels also enhanced the wound healing process. The mechanical properties of the hydrogels were enhanced by combining sodium alginate with synthetic polymers or by using plasticizers. The films and nanofibers, on the other hand, were also effective as potential wound dressings for the treatment of burns.

Addition of antibacterial agents onto the films enhanced their antibacterial activity revealing their potential as wound dressings that can protect the wound from bacterial infections. Films are not useful for wounds with excessive exudates because of their poor thickness. Several sodium alginate-based films thicknesses were increased by crosslinking with selected polymers, resulting in their ability to absorb a large quantity of exudates. Films application in vivo revealed accelerated re-epithelization, and furthermore, the combination of laser therapy with sodium alginate-based film further enhanced the formation of blood vessels and improved collagen deposition. The process parameters and the ratio of polymers used in the preparation of nanofibres influence the properties of the nanofibers such as the average diameter, the morphology, smoothness, pore size, tensile strength, water vapour transmission rate, moisture permeability, etc. Comparing the prepared nanofibers with the commercially available wound dressing revealed that the inhibitory effect of the prepared nanofibers on cytokine production was higher when compared to the commercially available dressings. The nanofibers enhanced wound healing, characterized by re-epithelization, vascularization, and the formation of hair follicles, and they act as an artificial skin on the wound region until the formation of a new tissue.

Alginate-based wafers are characterized by flexible, soft, and non-brittle nature. The porosity of the wafers can be enhanced by annealing. However, sterilization of wafers by steam can be detrimental, thus leading to structural collapse due to moisture. On the other hand, gamma irradiation is the suitable method of sterilization of wafers at an appropriate dose. Despite several reports on the application of sodium alginate in wound dressing, there is only one recent research report on the application of sodium alginate in a topical formulation for wound dressing. The topical formulation reduced the wound area significantly in vivo. Although alginate is a useful material for the preparation of wound dressings, there is also a need to select appropriate reagents, initiators that would not result in cross-linking that can cause toxicity and hinder gaseous permeability. Alginate-based materials are currently used clinically in wound healing applications. The development of future dressings containing bioactive agents is likely to play a much more effective role in the management of wounds.

## Figures and Tables

**Table 1:** Barriers to effective treatment of wounds.

Barriers	Examples
Educational factor	Poor quality of research, lack of appropriate training, ritualistic practice and lack of appropriate skills.
Organizational factor	Lack of standardisation of practice that is acceptable, lack of expert opinion, instability in the health services.
Clinical factor	Bacterial infection, hypersensitivity, malnutrition, poor tissue perfusion, copious exudate, too much or too little information on wound management.
Psychosocial factor	Social isolation resulting in depression and reduced motivation with treatment, pain resulting in loss of sufficient sleep and lack of self-care.

**Table 2:** Commercially available alginate-based dressings.

Commercially Available Alginate-Based Wound Dressings	Composition	Applications	References
Algicell™	Sodium alginate, 1.4% silver	Diabetic foot ulcer, leg ulcers, pressure ulcers, donor sites, and traumatic and surgical wounds.	[25,35]
AlgiSite M™	Calcium alginate	Leg ulcers, pressure ulcers, diabetic foot ulcers and surgical wounds.	[36]
Comfeel Plus™	Sodium carboxymethylcellulose and calcium alginate	Ulcers such as venous leg ulcers, pressure ulcers; burns, donor sites, postoperative wounds and necrotic wounds.	[37]
Kaltostat™	Sodium alginate	Pressure ulcers, venous ulcers, diabetic ulcers, donor sites, and traumatic wounds.	[38]
Sorbsan™	Calcium alginate	Arterial, venous, and diabetic leg ulcers Pressure ulcers, post-operative wounds, donor and graft sites and traumatic wounds.	[39]
Tegagen™	Sodium alginate	Diabetic and infected wounds.	[40]
Guardix-SG^®^	Sodium alginate and poloxamer	To avoid post-operative adhesions in thyroid and spine surgeries.	[34]
SeaSorb^®^	Calcium alginate	Good for high exuding wounds e.g., ulcers such as diabetic and leg pressure ulcers.	[34]
Algivon^®^	Calcium alginate and Manuka honey	It eliminates odour and ideal for necrotic wounds and wounds with odours.	[41]
Fibracol^TM^Plus	Calcium alginate and collagen	Full and partial-thickness wounds, for ulcers such as pressure ulcers, venous ulcers, diabetic ulcers and second-degree burns.	[42]
Hyalogran^®^	An ester of hyaluronic acid and sodium alginate	Used for ulcers, diabetic wounds, pressure sores, ischemic, necrotic wounds.	[43]
Tromboguard^®^	Sodium alginate, calcium alginate, chitosan, polyurethane and silver cations	Used to stop bleeding in postoperative wounds, traumatic wounds, gun shots, skin graft donor sites, bleeding from accidents.	[44]

## References

[1] Wound Classification. http://www.clinimed.co.uk/Wound-Care/Education/Wound-Essentials/Wound-Classification.aspx.

[2] Baranoski S., Ayello E.A., Langemo D.K., Mills J.E. (2008). Wound assessment. Wound Care Essentials: Practice Principles.

[3] Cullum N., Buckley H., Dumville J., Hall J., Lamb K., Madden M., Morley R., O’Meara S., Goncalves P.R., Soares M. (2016). Wounds research for patient benefit: A 5-year programme of research. https://www.ncbi.nlm.nih.gov/books/NBK379923.

[4] Frykberg R.G., Banks J. (2015). Challenges in the treatment of chronic wounds. Adv Wound Care (New Rochelle).

[5] Schmidtchen A., Pang C., Ni G., Sönnergren H., Car J., Järbrink K., Bajpai R. (2017). The humanistic and economic burden of chronic wounds: A protocol for a systematic review. Systematic reviews. Syst. Rev..

[6] Flanagan M. Barriers to the Implementation of Best Practice in Wound Care. http://www.wounds-uk.com/pdf/content_9030.pdf.

[7] Yadav P., Yadav H., Shah V.G., Shah G., Dhaka G. (2015). Biomedical biopolymers, their origin and evolution in biomedical sciences: A systematic review. J. Clin. Diagn. Res..

[8] Ulery B.D., Nair L.S., Laurencin C.T. (2011). Biomedical applications of biodegradable polymers. J. Polym. Sci. Pol. Phys..

[9] Sudarsan S., Franklin D.S., Guhanathan S. (2015). Imbibed salts and pH-responsive behaviours of sodium-alginate based eco-friendly biopolymeric hydrogels-A solventless approach. MMAIJ.

[10] Dhivya S., Padma V.V., Santhini E. (2015). Wound dressings—A review. BioMedicine.

[11] Sood A., Granick M.S., Tomaselli N.L. (2014). Wound dressings and comparative effectiveness data. Adv. Wound Care.

[12] Sezer A.D., Cevher E., Pignatello R. (2011). Biopolymers as wound healing materials: Challenges and new strategies. Biomaterials Applications for Nanomedicine.

[13] Zahedi P., Rezaeian I., Ranaei-Siadat S.O., Jafari S.H., Supaphol P. (2010). A review on wound dressings with an emphasis on electrospun nanofibrous polymeric bandages. Polym. Adv. Technol..

[14] Siddiqui A.R., Bernstein J.M. (2010). Chronic wound infection: Facts and controversies. Clin. Dermatol..

[15] Demidova-Rice T.N., Hamblin M.R., Herman I.M. (2012). Acute and impaired wound healing: Pathophysiology and current methods for drug delivery, Part 1: Normal and chronic wounds: Biology, causes, and approaches to care. Adv. Skin Wound Care.

[16] Cowan L., Stechmiller J., Phillips P., Schultz G., Krasner D.L., Rodeheaver G.T., Sibbald R.G., Woo K.Y. (2012). Science of wound healing: Translation of bench science into advances for chronic wound care. Chronic Wound Care: A Clinical Source Book for Healthcare Professionals.

[17] Benbow M. (2016). Best practice in wound assessment. Nurs. Stand..

[18] Eming S.A., Martin P., Tomic-Canic M. (2014). Wound repair and regeneration: Mechanisms, signaling, and translation. Sci. Transl. Med..

[19] Sun B.K., Siprashvili Z., Khavari P.A. (2014). Advances in skin grafting and treatment of cutaneous wounds. Science.

[20] Aller M.A., Arias J.I., Arraez-Aybar L.A., Gilsanz C., Arias J. (2014). Wound healing reaction: A switch from gestation to senescence. World J. Exp. Med..

[21] Zuliani-Alvarez L., Midwood K.S. (2015). Fibrinogen-related proteins in tissue repair: How a unique domain with a common structure controls diverse aspects of wound healing. Adv. Wound Care.

[22] Guo S.A., DiPietro L.A. (2010). Factors affecting wound healing. J. Dental Res..

[23] Sheikh Z., Hamdan N., Ikeda Y., Grynpas M., Ganss B., Glogauer M. (2017). Natural graft tissues and synthetic biomaterials for periodontal and alveolar bone reconstructive applications: A review. Biomater. Res..

[24] Oro F.B., Sikka R.S., Wolters B., Graver R., Boyd J.L., Nelson B., Swiontkowski M.F. (2011). Autograft versus allograft: An economic cost comparison of anterior cruciate ligament reconstruction. Arthroscopy.

[25] Lee K.Y., Mooney D.J. (2012). Alginate: Properties and biomedical applications. Prog. Polym. Sci..

[26] Fertah M., Belfkira A., Taourirte M., Brouillette F. (2017). Extraction and characterization of sodium alginate from Moroccan Laminaria digitata brown seaweed. Arab. J. Chem..

[27] Ching S.H., Bansal N., Bhandari B. (2017). Alginate gel particles—A review of production techniques and physical properties. Crit. Rev. Food Sci. Nutr..

[28] Donati I., Paoletti S. (2009). Material properties of alginates. Alginates: Biology and applications.

[29] Yang C.H., Wang M.X., Haider H., Yang J.H., Sun J.-Y., Chen Y.M., Zhou J., Suo Z. (2013). Strengthening alginate/polyacrylamide hydrogels using various multivalent cations. ACS Appl. Mat. Interf..

[30] Draget K.I., Taylor C. (2011). Chemical, physical and biological properties of alginates and their biomedical implications. Food Hydrocoll..

[31] Topuz F., Henke A., Richtering W., Groll J. (2012). Magnesium ions and alginate do form hydrogels: A rheological study. Soft Matter.

[32] Mørch Y.A., Qi M., Gundersen P.O., Formo K., Lacik I., Skjåk-Bræk G., Oberholzer J., Strand B.L. (2012). Binding and leakage of barium in alginate microbeads. J. Biomed. Mater. Res. A.

[33] Yin M., Xu F., Ding H., Tan F., Song F., Wang J. (2015). Incorporation of magnesium ions into photo-crosslinked alginate hydrogel enhanced cell adhesion ability. J. Tissue Eng. Regen. Med..

[34] Szekalska M., Puciłowska A., Szymańska E., Ciosek P., Winnicka K. (2016). Alginate: Current Use and Future Perspectives in Pharmaceutical and Biomedical Applications. Int. J. Polym. Sci..

[35] ALGICELL® Ag Antimicrobial Alginate Dressing. http://www.woundsource.com/product/algicell-ag-antimicrobial-alginate-dressing.

[36] AlgiSite M™. http://www.smith-nephew.com/professional/products/advanced-wound-management/algisite-m/.

[37] SMTL Dressings Datacard. http://www.dressings.org/Dressings/comfeel-plus.html.

[38] KALTOSTAT Calcium Sodium Alginate Dressing. https://fsastore.com/KALTOSTAT-Calcium-Sodium-Alginate-Dressing-3-x-4-34-Box-of-10-P23356.aspx.

[39] Sorbsan Flat. http://www.aspenmedicaleurope.com/specialist_wound_car/sorbsan-flat/.

[40] O’Meara S., Martyn-St James M., Adderley U.J. Alginate Dressings for Venous Leg Ulcers. http://eprints.whiterose.ac.uk/92499/1/O%27Meara_et_al-2015-The_Cochrane_Library.pdf.

[41] Algivon. http://www.advancis.co.uk/products/activon-manuka-honey/algivon.

[42] FIBRACOL™ Plus Collagen Wound Dressing with Alginate. http://www.woundsource.com/product/fibracol-plus-collagen-wound-dressing-alginate.

[43] HYALOGRAN® Biodegradable Wound Dressing. http://www.anikatherapeutics.com/products/dermal/hyalogran/.

[44] Tromboguard®. http://matopat.ro/wp-content/uploads/sites/2/2013/12/tromboguard-leaflet.pdf.

[45] Almeida J.F., Ferreira P., Lopes A., Gil M.H. (2011). Photocrosslinkable biodegradable responsive hydrogels as drug delivery systems. Int. J. Biol. Macromol..

[46] Huang S., Fu X. (2010). Naturally derived materials-based cell and drug delivery systems in skin regeneration. J. Control. Release.

[47] Jagur-Grodzinski J. (2010). Polymeric gels and hydrogels for biomedical and pharmaceutical applications. Polym. Adv. Technol..

[48] Sikareepaisan P., Ruktanonchai U., Supaphol P. (2011). Preparation and characterization of asiaticoside-loaded alginate films and their potential for use as effectual wound dressings. Carbohydr. Polym..

[49] Kamoun E.A., Kenawy E.R., Chen X. (2017). A review on polymeric hydrogel membranes for wound dressing applications: PVA-based hydrogel dressings. J. Adv. Res..

[50] Hess C.T. (2002). Clinical Guide: Wound Care.

[51] Saarai A., Sedlacek T., Kasparkova V., Kitano T., Saha P. (2012). On the characterization of sodium alginate/gelatine-based hydrogels for wound dressing. J. Appl. Polym. Sci..

[52] Saarai A., Kasparkova V., Sedlacek T., Sáha P. A comparative study of crosslinked sodium alginate/gelatin hydrogels for wound dressing. In Proceeding of the 4th WSEAS International Conference on Energy and Development.

[53] Straccia M.C., d’Ayala G.G., Romano I., Oliva A., Laurienzo P. (2015). Alginate hydrogels coated with chitosan for wound dressing. Mar. Drugs.

[54] Yu W., Jiang Y.Y., Sun T.W., Qi C., Zhao H., Chen F., Shi Z., Zhu Y.J., Chen D., He Y. (2016). Design of a novel wound dressing consisting of alginate hydrogel and simvastatin-incorporated mesoporous hydroxyapatite microspheres for cutaneous wound healing. RSC Adv..

[55] Mohandas A., Sudheesh Kumar P.T., Raja B., Lakshmanan V.K., Jayakumar R. (2015). Exploration of alginate hydrogel/nano zinc oxide composite bandages for infected wounds. Int. J. Nanomed..

[56] Singh R., Singh D. (2012). Radiation synthesis of PVP/alginate hydrogel containing nanosilver as wound dressing. J. Mater. Sci. Mater. Med..

[57] Nazeri S., Ardakani E.M., Babavalian H., Latifi A.M. (2015). Evaluation of Effectiveness of Honey-Based Alginate Hyrogel on Wound Healing in a Mouse Model of Rat. J. Appl. Biotechnol. Rep..

[58] Murakami K., Aoki H., Nakamura S., Nakamura S.I., Takikawa M., Hanzawa M., Kishimoto S., Hattori H., Tanaka Y., Kiyosawa T. (2010). Hydrogel blends of chitin/chitosan, fucoidan and alginate as healing-impaired wound dressings. Biomaterials.

[59] Kamoun E.A., Kenawy E.R., Tamer T.M., El-Meligy M.A., Eldin M.S. (2015). Poly (vinyl alcohol)-alginate physically crosslinked hydrogel membranes for wound dressing applications: Characterization and bio-evaluation. Arab. J. Chem..

[60] Xing N., Tian F., Yang J., Li Y.K. (2012). Characterizations of Alginate-Chitosan Hydrogel for Wound Dressing Application. Adv. Mater. Res..

[61] Rudyardjo D.I., Wijayanto S. (2017). The synthesis and characterization of hydrogel chitosan-alginate with the addition of plasticizer lauric acid for wound dressing application. J. Phys. Conf. Ser..

[62] Devi M.P., Sekar M., Chamundeswari M., Moorthy A., Krithiga G., Murugan N.S., Sastry T.P. (2012). A novel wound dressing material-fibrin-chitosan-sodium alginate composite sheet. Bull. Mater. Sci..

[63] Zhou Z., Chen J., Peng C., Huang T., Zhou H., Ou B., Chen J., Liu Q., He S., Cao D. (2014). Fabrication and physical properties of gelatin/sodium alginate/hyaluronic acid composite wound dressing hydrogel. J. Macromol. Sci. Part A.

[64] Liakos I., Rizzello L., Scurr D.J., Pompa P.P., Bayer I.S., Athanassiou A. (2014). All-natural composite wound dressing films of essential oils encapsulated in sodium alginate with antimicrobial properties. Int. J. Pharm..

[65] Pereira R., Carvalho A., Vaz D.C., Gil M.H., Mendes A., Bártolo P. (2013). Development of novel alginate based hydrogel films for wound healing applications. Int. J. Biol. Macromol..

[66] Pereira R., Tojeira A., Vaz D.C., Mendes A., Bártolo P. (2011). Preparation and characterization of films based on alginate and *Aloe vera*. Int. J. Polym. Anal. Charact..

[67] George M., Joseph L., Francis L.T. (2017). Development and evaluation of silver sulphadiazine loaded sodium alginate gelatin film for wound dressing applications. Eur. J. Pharm. Med. Res..

[68] Li S., Li L., Guo C., Qin H., Yu X. (2017). A promising wound dressing material with excellent cytocompatibility and proangiogenesis action for wound healing: Strontium loaded Silk fibroin/Sodium alginate (SF/SA) blend films. Int. J. Biol. Macromol..

[69] Rezvanian M., Amin M.C., Ng S.F. (2016). Development and physicochemical characterization of alginate composite film loaded with simvastatin as a potential wound dressing. Carbohydr. Polym..

[70] Thu H.E., Zulfakar M.H., Ng S.F. (2012). Alginate based bilayer hydrocolloid films as potential slow-release modern wound dressing. Int. J. Pharm..

[71] Dantas M.D., Cavalcante D.R., Araújo F.E., Barretto S.R., Aciole G.T., Pinheiro A.L., Ribeiro M.A., Lima-Verde I.B., Melo C.M., Cardoso J.C. (2011). Improvement of dermal burn healing by combining sodium alginate/chitosan-based films and low level laser therapy. J. Photochem. Photobiol. B Biol..

[72] Sharma S., Sanpui P., Chattopadhyay A., Ghosh S.S. (2012). Fabrication of antibacterial silver nanoparticle-sodium alginate-chitosan composite films. RSC. Adv..

[73] Abrigo M., McArthur S.L., Kingshott P. (2014). Electrospun nanofibers as dressings for chronic wound care: Advances, challenges, and future prospects. Macromol. Biosci..

[74] Zhang Y., Lim C.T., Ramakrishna S., Huang Z.M. (2005). Recent development of polymer nanofibers for biomedical and biotechnological applications. J. Mater. Sci. Mater. Med..

[75] Dahlin R.L., Kasper F.K., Mikos A.G. (2011). Polymeric nanofibers in tissue engineering. Tissue Eng. Part B Rev..

[76] Andreu V., Mendoza G., Arruebo M., Irusta S. (2015). Smart dressings based on nanostructured fibers containing natural origin antimicrobial, anti-inflammatory, and regenerative compounds. Materials.

[77] Shalumon K.T., Anulekha K.H., Nair S.V., Nair S.V., Chennazhi K.P., Jayakumar R. (2011). Sodium alginate/poly (vinyl alcohol)/nano ZnO composite nanofibers for antibacterial wound dressings. Int. J. Biol. Macromol..

[78] Hu C., Gong R.H., Zhou F.L. (2015). Electrospun sodium alginate/polyethylene oxide fibers and nanocoated yarns. Int. J. Polym. Sci..

[79] Hajiali H., Summa M., Russo D., Armirotti A., Brunetti V., Bertorelli R., Athanassiou A., Mele E. (2016). Alginate–lavender nanofibers with antibacterial and anti-inflammatory activity to effectively promote burn healing. J. Mater. Chem. B.

[80] Üstündağ G.C., Özbek S., Karaca E., Çavuşoğlu İ. (2010). In vivo evaluation of electrospun poly (vinyl alcohol)/sodium alginate nanofibrous mat as wound dressing. Tekstil ve Konfeksiyon.

[81] Coşkun G., Karaca E., Ozyurtlu M., Özbek S., Yermezler A., Çavuşoğlu İ. (2014). Histological evaluation of wound healing performance of electrospun poly (vinyl alcohol)/sodium alginate as wound dressing in vivo. BioMed. Mater. Eng..

[82] Park S.A., Park K.E., Kim W. (2010). Preparation of sodium alginate/poly (ethylene oxide) blend nanofibers with lecithin. Macromol. Res..

[83] Fu R., Li C., Yu C., Xie H., Shi S., Li Z., Wang Q., Lu L. (2016). A novel electrospun membrane based on moxifloxacin hydrochloride/poly (vinyl alcohol)/sodium alginate for antibacterial wound dressings in practical application. Drug Deliv..

[84] Lee S.M., Park I.K., Kim Y.S., Kim H.J., Moon H., Mueller S., Jeong Y.I. (2016). Physical, morphological, and wound healing properties of a polyurethane foam-film dressing. Biomater. Res..

[85] Foam Dressings. https://woundeducators.com/foam-dressings.

[86] Hegge A.B., Andersen T., Melvik J.E., Bruzell E., Kristensen S., Tønnesen H.H. (2011). Formulation and bacterial phototoxicity of curcumin loaded alginate foams for wound treatment applications: Studies on curcumin and curcuminoides XLII. J. Pharm. Sci..

[87] Valerón Bergh V.J., Johannessen E., Andersen T., Tønnesen H.H. (2017). Evaluation of porphyrin loaded dry alginate foams containing poloxamer 407 and β-cyclodextrin-derivatives intended for wound treatment. Pharm. Dev. Technol..

[88] Topical Semi-Solid Dosage Forms. https://www.malvern.com/en/industry-applications/sample-type-form/topicals-creams-and-gels.

[89] (2015). Topical Delivery—The Importance of the Right Formulation in Topical Drug Development. http://www.drug-dev.com/Main/Back-Issues/TOPICAL-DELIVERY-The-Importance-of-the-Right-Formu-833.

[90] Ahmed M.M., Jahangir M.A., Saleem M.A., Kazmi I., Bhavani P.D., Muheem A. (2015). Formulation and Evaluation of Fucidin Topical Gel Containing Wound Healing Modifiers. Am. J. PharmTech Res..

[91] Fredric S., Gowda D.V., Yashashwini M. (2015). Wafers for wound healing. J. Chem. Pharm. Res..

[92] Lipsky B.A., Hoey C. (2009). Topical antimicrobial therapy for treating chronic wounds. Clin. Infect. Dis..

[93] Boateng J., Burgos-Amador R., Okeke O., Pawar H. (2015). Composite alginate and gelatin based bio-polymeric wafers containing silver sulfadiazine for wound healing. Int. J. Biol. Macromol..

[94] Matthews K.H., Stevens H.N., Auffret A.D., Humphrey M.J., Eccleston G.M. (2005). Lyophilised wafers as a drug delivery system for wound healing containing methylcellulose as a viscosity modifier. Int. J. Pharm..

[95] Pawar H.V., Boateng J.S., Ayensu I., Tetteh J. (2014). Multifunctional medicated lyophilised wafer dressing for effective chronic wound healing. J. Pharm. Sci..

[96] Gowda D.V., Fredric S., Srivastava A., Osmani R.A. (2016). Design and development of antimicrobial wafers for chronic wound healing. Pharm. Lett..

